# Preschoolers consider (absent) others when choosing a distribution procedure

**DOI:** 10.1371/journal.pone.0221186

**Published:** 2019-08-29

**Authors:** Patricia Grocke, Federico Rossano, Michael Tomasello

**Affiliations:** 1 Department of Developmental and Comparative Psychology, Max-Planck-Institute for Evolutionary Anthropology, Leipzig, Germany; 2 Department of Cognitive Science, University of California, San Diego, California, United States of America; Mälardalen University, SWEDEN

## Abstract

This study investigated how the presence of others and anticipated distributions for self influence children’s fairness-related decisions in two different socio-moral contexts. In the first part, three- and five-year-old children (N = 120) decided between a fair and an unfair wheel of fortune to allocate resources (procedural justice). In the second part, they directly chose between two distributions of resources (distributive justice). While making a decision, each child was either observed by the affected group members (public), alone (private), or no others were introduced (non-social control). Children choose the fair option more often when others were affected (independently of their presence) only in the procedural justice task. These results suggest that using a fair procedure to distribute resources allows young preschoolers to overcome selfish tendencies.

## Introduction

In our daily lives, we are often confronted with situations in which we have to decide whether we want to follow selfish or pro-social motives. It is obvious why we sometimes give ourselves the biggest piece of the cake. However, it is less clear what makes us engage in fair behaviors by sharing our resources and giving up advantages in the interest of others. By studying children, we can understand how and when selfish motives can be overcome and fairness concerns develop. Splitting up a resource equally is one way to achieve fairness (distributive justice). If this is not possible, fairness can be achieved by splitting up the chance of getting the biggest part of the share by using a fair decision-making procedure (procedural justice). While there is a wealth of studies on the development of distributive justice concerns, the investigation of procedural justice concerns in children has received less attention and the relation between these two basic kinds of fairness is still unknown.

Shortly after their first birthday infants begin to recognize unfair distributions of resources [1]. In the second year of life they anticipate resources to be distributed equally, prefer agents doing so and expect them to be rewarded for such fair behavior [2–4]. Schmidt and Sommerville[5] showed that there is a relation between children’s fairness judgements and their actual sharing behavior at that early age. Two-year-olds have been shown to share resources equally not only with adults but also with peers [6]. However, by the age of three a phenomenon called the ‘knowledge-behavior gap’ occurs: Children of that age state that they should share a given resource equally with others, although they actually fail to do so until early school age [7, 8]. This is in line with other studies showing that preschoolers are more likely to make fair decisions in a third-party paradigm than when their own outcome was affected [9–11]. Moreover, studies investigating inequity aversion show that preschoolers reject resource distributions which are disadvantageous for themselves but do not do so when it is advantageous for them and the other party gets less [12,13]. In this period between the age of three and five years the child’s prosocial motivation clashes with her self-serving tendencies and further investigation of children in this age range would be of special interest for research on the development of their sense for fairness.

Several paradigms have been used to study children’s willingness to engage in costly sharing. One that might be most appropriate for young preschoolers in particular is the mini-dictator game, in which children can choose between two options–a fair one, with an equal distribution of resources and an unfair one favoring themselves. Some studies using this paradigm found an increased tendency to pick the fair option in the late preschool years [14–16]others found no difference between three- and six-year-olds [17, 18], and two studies even found decreasing rates of fair choices within that age range [19, 20]. Interestingly, the latter two studies were the only ones in which peer receivers were present and watching the proposers making their choices. This appears illogical since in theory reputational concerns would predict making a fair choice in the presence of the other affected parties and giving up an advantage for the sake of the group expecting direct reciprocity. Such behavior could establish a positive reputation by signaling that the individual is a good cooperator [21]—preschoolers already seem to know that and act accordingly when donating resources comes at no cost to them [22, 23]. However, in costly sharing situations feelings like envy and spite trump their prosocial tendencies [24, 25]. Assuming that reputation management is a driving force in children’s prosocial development, another neglected feature of sharing situations needs to be considered: the number of parties affected by a decision. The typical set-up for studies on costly sharing are dyadic situations in which the child is asked to share with one other individual and sometimes this individual is not even aware of the decision. But this only partially depicts the situations we face in our every-day life in which we often have to attune the interests of several other individuals or groups. Furthermore, the development of fairness concerns can be understood as a progress starting with rather egocentric, selfish children who become more and more willing to give some of their resources to others until they finally reach the fair equal split. The dichotomy between fair (maximal donation without being disadvantaged) and unfair (any other donation including zero) might not do justice to this progress. Former studies possibly underestimated children’s fairness concerns in costly sharing situations. We provide a new design which 1. emphasizes the social significance of their decision by using a group context, 2. reduces the probability that envy obscures their prosocial motivation by providing a prosocial option with a minimal loss for the donors outcome and 3. assesses whether children have a binary sense of fairness (either everybody gets the same or not) or whether distributive justice can be scalar, in the sense that there might be different degrees of unfairness and they can do something to make a situation less unfair.

Recently another aspect of fairness has gained some attention in the field: the understanding and use of fair decision making procedures. A fair procedure provides every participating party with the same chance of getting a certain resource. In their late preschool years, children begin to prefer a fair procedure over an unfair one (favoring one party) when allocating resources among two other agents [26]. But also in a first-person scenario [27], in which children decide together as a group and are both decider and receiver, they accept unequal resource distributions if a fair decision making procedure has been deployed and they reject an unfair procedure, giving one group member an advantage. This could be interpreted as an honest concern for equality of opportunities, which seemingly develops even before children come to a clear preference for equality of resource distributions. However, in this study, a group of three children had to negotiate whether to use a certain procedure (fair or unfair) to distribute reward packages or not. When confronted with an unfair procedure, the majority of the group members were disadvantaged (two of the three children). Another possible explanation would therefore be that the two disadvantaged group members just hindered the advantaged one from acting selfishly. A different study design is needed to investigate whether and when children would take a cost to reject unfair decision-making procedures in the interest of others. Furthermore the youngest subjects in the reported studies on procedural justice were five years old. While research on distributive justice covers various ages beginning in infancy, the developmental trajectory of young children’s sensitivity for procedural justice has not been investigated so far. Procedural justice is one (if not the) important quality of many of the implicit and explicit rules which govern our social life [28]. Many of them aim at preventing the individual from taking advantage of others or in other words giving priority to self-interest over the common good. It might be easier to follow such an equalizing rule or procedure than willingly giving up resources in the interest of others. If procedural justice does have such a regulating force, it might also help young children to overcome their self-serving tendencies even before they engage in costly equal sharing.

We therefore designed a study presenting 3- and 5-year-old children with two situations in which they had to make a decision for all members of their group (therefore including themselves): One was choosing one of two wheels of fortune to assign unequal reward packages to the group members (procedural justice). The other was choosing one of two reward distributions (distributive justice). In both cases, the children could choose between an unfair option favoring themselves and a second option requiring them to give up an advantage in the interest of their group.

We manipulated the presence of the other receivers in three conditions. In the public condition, the test children made their choices in front of two other group members. In the private condition, the other group members left the room prior to the child’s decision. We also included a non-social control condition in which the child was alone and no other group members were introduced.

This novel study design allowed us to investigate young children’s distributive and procedural justice concerns in parallel and with regard to the effect of the presence of affected others. The latter is an important factor that varied across previous studies on costly sharing but has not been systematically manipulated within a study before. We furthermore isolated an individual’s decision within a group context, while former work either studied individual decisions within a dyadic interaction or decisions made by a group as a whole. This is especially relevant when studying procedural justice since complex decision-making procedures only become necessary when more straightforward distributive norms and rules cannot be applied. However, the procedure was still simple enough to be understood by three-year-old preschoolers which extends the age range in which procedural justice could be investigated.

If reputation management has an influence on children’s decision making, they should make more prosocial choices when the affected group members are present than when they are not watching. If decision making procedures help children overcome self-serving tendencies in costly sharing situations, three-year-olds should already be able to make more fair choices in the social conditions compared to the non-social control condition–but only in the procedure choice task and not in the distribution choice task. Making fair decisions is a complex behavior based on many different factors—some of which we investigate in this study.

## Method

### Participants

We tested 120 participants (60 female) from two age groups (M = 5 years, 6 months, SD = 1 month, M = 3 years, 7 months, SD = 2 months) in a between-subjects design. The sample size was specified prior to data collection, based on typical sample sizes in this field. Children were randomly assigned to one of the three conditions. The children came from mixed socio-economic backgrounds and were recruited via urban daycare centers (where testing also took place).

Eighteen additional children were tested but excluded because the child failed to answer the first (procedure choice test) (7), second (distribution choice test) (6) or both comprehension control questions correctly (4), or because the child misunderstood the procedure (1). In one of the three conditions (public), the child was grouped with two same-sex peers who acted as confederates (for the three-year-old participants: M = 4 years, 8 months, SD = 3 months; for the five-year-old participants: M = 5 years, 9 months, SD = 2 months).

In both group conditions, there were groups in which the tested child was familiar with both play partners (private: 25, public: 13) or one of the play partners (private: 11, public: 10) because they were from the same kindergarten group; but there were also groups consisting entirely of strangers (private: 4, public: 7). This study was approved by the Max Planck Institute for Evolutionary Anthropology Child Subjects Committee on June 10, 2015. It was carried out with the written informed consent of the participants, and in accordance with all applicable laws and rules governing psychological research in Germany.

### Material and procedure

Testing was conducted by two experimenters. The instructing experimenter (E1) explained the game to the children and asked the actual test questions. The other experimenter (E2) manipulated the apparatus and instructed the confederates.

Each child went through a warm-up phase and two test situations. The two experimenters picked the children up from their kindergarten groups and brought them to the testing room. First, the children played a warm-up game to get to know the apparatus used in the first testing situation (wheel of fortune). This was followed by three test trials in which one of the children (actor) had to choose one of two wheels (procedure choice situation). Then E1 introduced the second test situation in which the actor had to choose one of two distributions of resources in three test trials.

#### Warm-up

**Fig 1:**
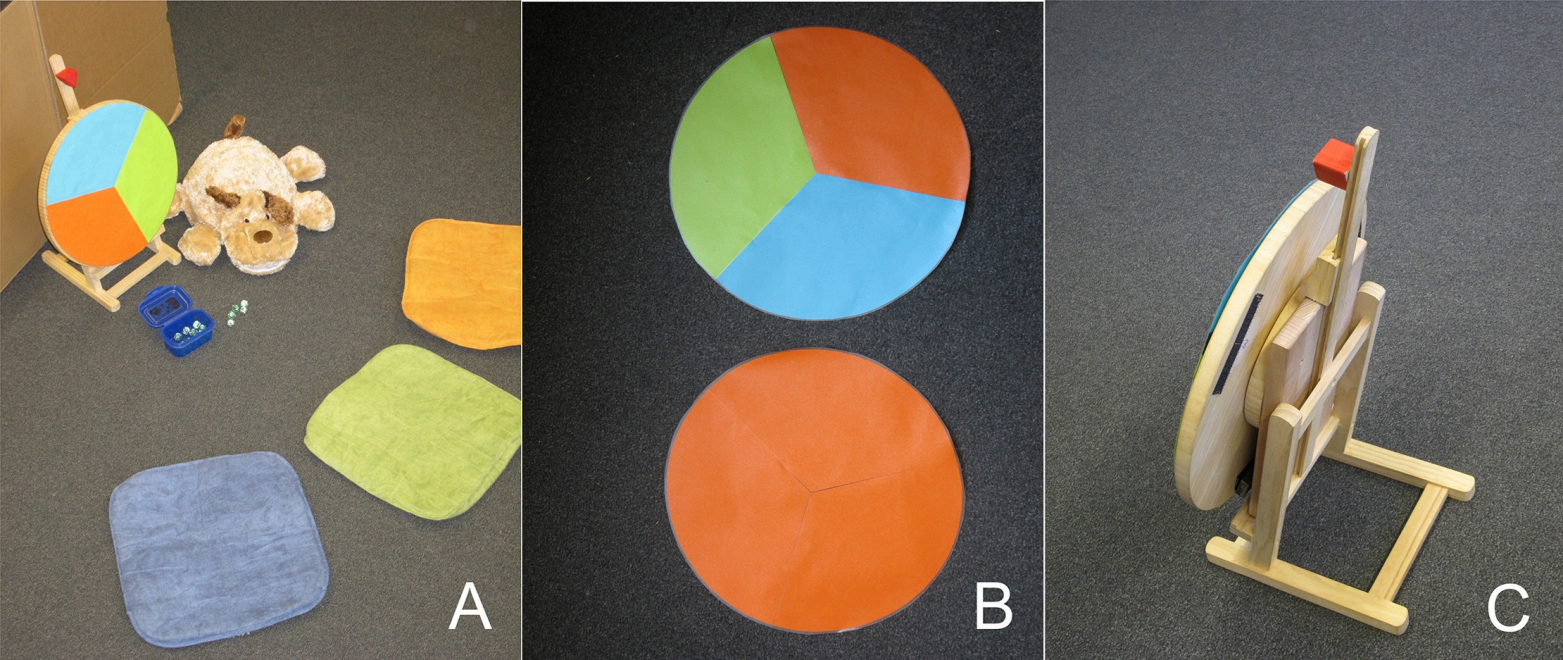
Material and set-up. (a) Set-up for the warm-up with colored cushions for the children to sit on, a fair wheel of fortune with each of the colors on it and the stuffed animal dog, (b) two wheel covers used in the test (fair–all three children could get the big sticker box, unfair: only the tested child sitting on the orange cushion could get the big sticker box), the chosen cover was attached to the wheel dummy and then the wheel was spun once, (c) back of the wheel with a weight attached to it. The weight could be fixed to three different positions on the back of the wheel to determine the final position after spinning.

After entering the test room, every child was assigned to one of three colored cushions (orange, blue, green) and told that the color of the cushion was their personal color for the games to come. A stuffed animal dog that had not eaten breakfast that morning and needed to be fed with marbles was introduced. A wheel of fortune was used to decide whose turn it was to feed the dog (Fig 1). The wheel stood upright on a stand. Exchangeable covers could be fixed to the front and a weight could be fixed to the back of the wheel to manipulate the final position of it after spinning. This went unnoticed by the children. After three rounds with a fair wheel (with all three colors) in which all children fed the dog once, the unfair wheels (one color) were introduced and one round was played with each of them.

#### Procedure choice test

**Fig 2:**
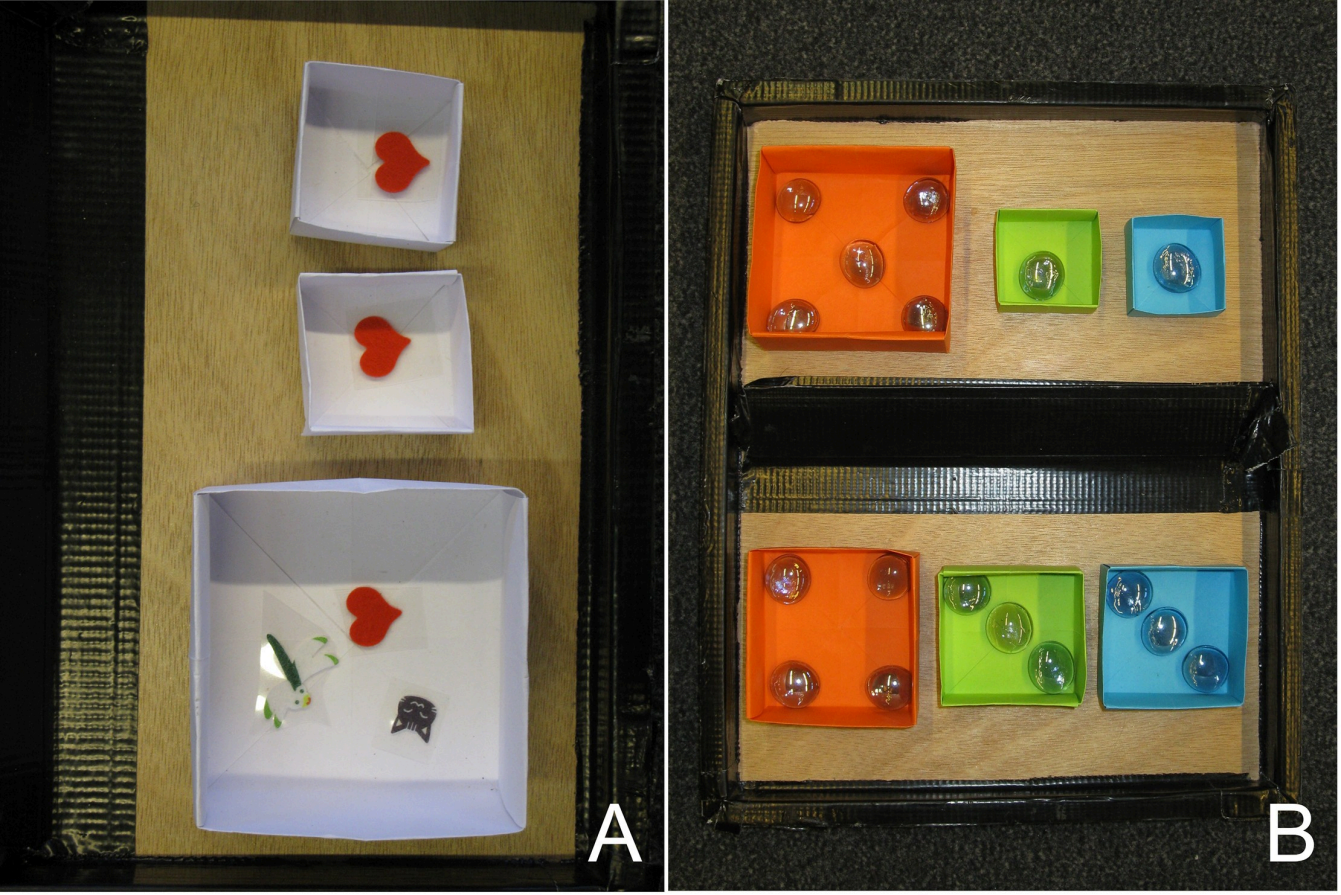
Material and set-up. (a) Boxes with reward stickers in the procedure choice game and (b) glass stones in the distribution choice game. The size of the boxes reflected the number of rewards they contained.

E1 showed three boxes containing different numbers of stickers (3, 1, 1) to the children (Fig 2A) and explained that each child could have one of the boxes and keep the stickers. But first they had to spin the wheel to decide who got the big box with three stickers. Then E1 put the fair wheel (same chance for all children) and an unfair wheel (favoring the tested child) in front of the child and asked: “Now we have to choose one wheel to decide who gets the big box. Which one should we use?” Then the wheel chosen by the child was placed on the wheel dummy and spun. It was not necessary to spin the wheel a second time, since it was only used to decide about the big sticker box. The two small sticker boxes were always given to the two children whose colors were not shown by the wheel. The wheel was never spun a second time within one trial. Furthermore, the wheel was manipulated so that an actor choosing the fair wheel never won in trial 1 or 2. This was necessary to make sure that previous successes do not influence the actor’s decision in the following trials and to keep the experience that different participants have with the wheel consistent. The stickers were collected in colored cups (orange, blue, green). After three trials, E1 said that the game was over and announced the second game.

#### Distribution choice test

For the second test situation, a tray with two rows of colored boxes was shown to the children. Each row consisted of an orange, a green and a blue box containing different numbers of glass stones (Fig 2B). We used glass stones instead of stickers like in the procedure choice task because we framed this second part of the experiment as a totally different and new game and wanted to prevent carryover effects. In the “rather fair” row, the distribution of stones was four for the participant and three for each of the other children. In the “unfair” row, the distribution was five for the participant and one for each of the other children. Note that in both options the participant had more rewards than the group members because we did not want the child to make unfair choices out of a preference for others getting less than herself [29]. Furthermore, we tested an age range (3-5yo) in which children are known to be fine with advantageous inequality when rejecting or accepting an offer (e.g., [12, 13]). Many of them keep the majority of resources for themselves in dictator games (e.g., [8]). In the task we wanted to create a situation in which we could investigate whether preschoolers, despite their overall selfish tendency, still consider their play partner’s outcome or the outcome of the group as a whole. The distribution in the options we chose (5-1-1 vs. 4-3-3) is the smallest possible version with two crucial features: a) the decider gets the biggest part of the share in both options and b) preferring the unfair over the fair option means a small gain for the decider and a relatively big loss for the two others. Hence, it is rather easy to be fair in our task compared to other studies.

Then the children were asked whether they remembered their color and which of the boxes were theirs. The rule of the game was that only one of the two rows of boxes could be taken and the children had to choose one. E1 started the game and asked the participant: “Now we have to choose one row with boxes. Which one?” Then the side not chosen by the child was covered and each child took her box from the chosen side. The glass stones were collected in colored cups. After three trials E1 said that the game was over and allowed the children to choose three glass stones out of a big ‘treasure chest’. Glass stones and stickers were put into envelopes so the children could take them home and the two experimenters brought the children back to their kindergarten groups.

#### Conditions

The children were tested in three different conditions in a between-subjects design. In the *public* condition the participant was grouped with two other children (confederates). After explaining the procedure choice game to all three children, E1 left the room under a pretext and asked the participant to come with her. E2 stayed with the confederates and trained them to keep quiet and let the participant decide by making them aware of and comfortable with the possibility that they would get only one sticker in all trials and promising them three additional stickers each (bigger and more attractive than the test stickers) if they followed the instructions. Then E1 came back with the participant and the game started. After the instructions for the distribution choice game, E2 who trained the confederates left the room and asked the two confederates to come with her. Outside the room, the children were rewarded for keeping quiet in the first game and were trained to behave in the same way in the second game. Again, E2 told them that it was possible that the participant might always take the unfair option, but that in the end they would be allowed to choose three glass stones from a treasure chest which they could take home anyway. Then they entered the test room again and played the second game.

In the *private* condition three children were instructed together as in the *public* condition. Right before the test, E2 left the room with two of the children under a pretext and E1 played the two games with one child, stating that they did not have to wait for the other two children to come back. The participant was asked to decide for the three of them instead. After the first participant finished playing the two test games, one of the waiting children came in and participated in the test, and after that so did the third one. In the meantime, the waiting children were kept busy by painting with E2 outside the test room, and not allowed to talk about the games, but in this case none of the children was instructed further by the experiment to act as a confederate, since each child was going to participate in the study separately.

In the *control* condition each participant was instructed alone, without any other child present in the room. In the warm-up, E1 explained that they would only feed the dog when the child’s color came up on the wheel. If one of the other colors came up no one would feed the dog because the colors did not belong to anyone and they would just spin it again. In the procedure choice test, the wheel was used to decide whether the child would get the big box with three stickers or one of the smaller ones with one sticker. After spinning the wheel and giving the participant her sticker box, E1 emptied the remaining boxes into the colored cups also used in the other conditions and said: “And these stickers don’t belong to anyone, we can put them away.” E1 proceeded the same way in the distribution choice task.

#### Comprehension control questions

After providing the instructions for each of the games, E1 asked the participant two questions to check whether she understood the two options to choose from. Before the procedure choice test the child was shown the fair and the unfair wheel of fortune and asked: “Which of the two wheels was the one with which you’ll always get the big sticker box? And with which of the wheels could the other children also get the big sticker box?” Before the distribution choice test, E1 asked the child: “Look at your boxes. On which row do you have more glass stones? And now look at the other children’s boxes. On which row do they have more glass stones?” Children who could not answer these questions correctly in one or both situations were excluded from the sample, because their later choice would not be based on full awareness of the two options presented. The comprehension control questions were asked in private when E1 was alone with the participant. After answering the comprehension control questions correctly, E1 and the child repeated together what each color meant in terms of resources allocation (i.e. the connection to the affected children).

In the *control* condition, E1 did not refer to other children but instead asked: “Which one is the wheel with which you will always get the big sticker box? And which one is the wheel with which you sometimes get the big sticker box and sometimes you do not?” and “Look at your boxes. On which row do you have more glass stones? And in the boxes with the other colors–on which side are there more glass stones?”

#### Counterbalancing

We counterbalanced the positions of the fair and unfair options in both tasks. In the procedure choice task, half of the children got the fair wheel presented to their left and the unfair wheel to their right, the other half the other way around. In the distribution choice task, for half of the children the fair option was presented above the unfair option, for the other half the unfair option was presented above the fair one. Furthermore, we counterbalanced the order of the two comprehension control questions for each task.

### Measurement and coding

All sessions were videotaped and coded by the first author. Twenty five percent of the video recordings were coded a second time for reliability by an assistant blind to the research hypothesis (κ = 1). The reliability cases were picked randomly but in equal shares from all six combinations of age group and condition. We coded which of the two options the child chose in each of the tests and every trial as a binary measure (fair choice = 1, unfair choice = 0).

## Results

For each test situation (i.e. procedure choice test, distribution choice test), we ran a generalized linear mixed model [30] with binomial error structure in which we included age group (3-year-olds, 5-year-olds), condition (public, private, control), counterbalancing (order of presentation of the two options), gender and trial as fixed effects. Furthermore we included the interaction of age group and condition. To control for individual differences, we included subject as random factor and trial within subject as random slope. Results are reported separately for each test situation.

### Procedure choice test

**Fig 3:**
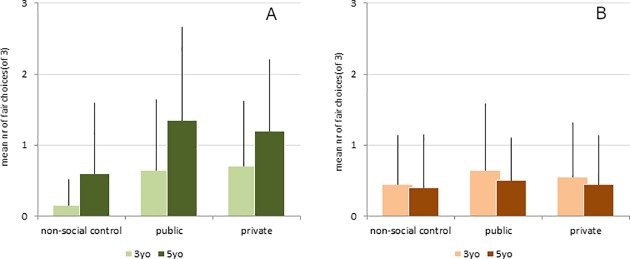
Results. Mean number and standard deviation of fair choices out of three trials (a) in the procedure choice test and (b) the distribution choice test.

Overall, the full model was significant (likelihood ratio test: χ^2^ = 22.3, df = 5, p< .001) in comparison to a null model (comprising only gender, the counterbalancing, the random effects and slope). We found no interaction between age group and condition (likelihood ratio test comparing a model including the interaction of age group and condition and a reduced model including no interaction (χ^2^ = 0.347, df = 2, p = .843)). Overall the five-year-old children made more fair choices than the three-year-old children (Z = 3.046, p = .002). Comparing the three conditions (Fig 3), children from both age groups chose the fair wheel more often in the *public* condition than in the *control* condition (Z = 2.994, p = .003) and also in the *private* condition compared to the *control* condition (Z = 2.922, p = .003). There was no difference between the *public* and the *private* condition (Z = 0.086, p = .932). We found no effect of gender (Z = -0.275, p = .783), counterbalancing (Z = 0.661, p = .509) or trial (Z = 0.191, p = .848).

### Distribution choice test

Overall, the full model was not significant (likelihood ratio test: χ^2^ = 1.364, df = 5, p = .928) in comparison to a null model (comprising only gender, the counterbalancing, the random effects and slope). Since our independent variables age group and condition had no effect on the children’s choices in the distribution choice test (Fig 3), we refrained from analyzing the data further.

### Additional analyses

Our study design included a non-social control condition which served as a baseline for the comparison with our two test conditions. This was our main analysis and is described above. Nevertheless, we additionally ran a set of exact binomial tests (see Table 1) to test the choices against chance. It revealed that children of both age groups in all three conditions of both tasks were significantly below chance level choosing the selfish option except for the 5-year-olds in the two social conditions of the procedure choice task (private, p = .155; public, p = .519).

**Table 1:** Proportion of fair choices out of 60 of both age groups (3- and 5-year-olds) in three conditions (control, private and public).

a) procedure choice task				b) distribution choice task			
	control	private	public		control	private	public
3-year-olds	5%***	21.67%***	23.33%***	3-year-olds	15%***	21.67%***	18.33%***
5-year-olds	20%***	45%	40%	5-year-olds	13.33%***	16.67%***	15%***

a) procedure choice task

b) distribution choice task

*** asterisks mark a significant deviance from random choice (result of exact binomial test)

In the *private* and the *public* condition, the groups differed with regard to the level of acquaintance among the children playing together (all strangers, target knew one play partner, target knew both play partners). We analyzed whether this had an influence on the children’s decision-making by running a generalized linear mixed model with binomial error structure including number of familiar play partners within the group (none, one, two), condition (public, private), age group (3-year-olds, 5-year-olds), counterbalancing (order of presentation of the two options), gender and trial as fixed effects. We also included subject as random factor and trial within subject as random slope. We ran one model for each test situation (procedure choice test and distribution choice test). The number of familiar play partners did not influence the children’s choices (procedure choice test: Z = -1.048, p = .295, distribution choice test: Z = 1.308, p = .191).

In the *private* condition, three children were introduced to the game together as a group, but were tested one after another with the others waiting outside. This might have led to order effects (e.g. the last child knowing that the other two already had their turn being less prosocial than the first). We analyzed whether this had an influence on the children’s decision-making by running a generalized linear mixed model with binomial error structure including order of testing (first, second, last), age group (3-year-olds, 5-year-olds), counterbalancing (order of presentation of the two options), gender and trial as fixed effects. We also included subject as random factor and trial within subject as random slope. We ran one model for each test situation (procedure choice test and distribution choice test). The order of testing did not influence the children’s choices in the distribution choice test (Z = -0.134, p = .893) but had a marginal effect in the procedure choice test (Z = -1.763, p = .078): The later it was their turn to play, the more likely the children were to choose the selfish option. The reason might possibly be that the social priming of the group interaction faded over time or the second and the third child realized that the other/s had their turn already and this would justify making selfish choices now that it was their turn to play. Hence, our approach to test all three children of the group one after the other in the *private* condition might have underestimated the children’s tendency to make prosocial choices. However, since we did not find any significant difference in their behavior compared to the *public* condition (in which we tested only one of the three participating children), we refrain from further interpretation of this marginal order effect.

## Discussion

We presented 3- and 5-year-old children with two situations in which they had to make a decision for their group (including themselves): One was choosing one of two wheels of fortune to assign unequal reward packages to the group members (procedural justice). The other was directly choosing one of two reward distributions in a mini-dictator game (distributive justice). In both situations the child could choose between an unfair option favoring herself and a prosocial option which was in the interest of the whole group. In the procedure choice test, both age groups chose the fair option more often when other group members were affected than when they were only deciding for themselves, no matter whether the others were present and aware of the decision or absent. However, in the distribution choice test, the children mostly chose the option favoring themselves without considering the other group members.

Deciding whether to keep an attractive resource for themselves or sharing it with others can be a difficult task for children. They know from very early on what would be the right thing to do, but they struggle to overcome their selfish motives [8]. In our distribution choice test, the child had to give up only one glass stone to provide two other children with two glass stones each. The outcome for the group as a whole would have been much bigger in the fair option and the participant would still have been the one with most glass stones compared to the other children. Compared to other studies, it was rather simple to be a fair player in our test, and yet the children mostly favored themselves and did not care about the other group members.

Since there was no equal distribution available and they had to choose between two degrees of unfairness, the children might have felt entitled to pick the most selfish option. This would suggest that preschoolers have a binary understanding of fairness with a distribution being either equal giving everybody the same or unequal including any other distribution of resources. A more mature understanding of fairness would be a scalar one with different degrees of unfairness depending on how big the difference between the various outcomes is. Assuming that this explanation accounts for the observed behavior, it would be interesting to frame the task differently in future studies, for example by letting the participant choose whether she wants to keep all of her rewards or would trade one of her rewards for four more rewards for the group (like in our task). In such a situation it is more obvious how little it would take to act in the interest of the group, which might lead to a higher willingness to make sacrifices. Future studies could also use a specific group marker like in the minimal group paradigm [31] to stress the cohesion within the group which might result in a higher rate of fair choices at least in the older age group [32]. In our task however, it might also have been the case that preschoolers are just not able or not willing to inhibit their selfish tendencies when directly confronted with their possible outcome [8, 33, 34]. This can lead to serious conflicts within a group.

One way to prevent such conflicts of interest within a group is to use a procedure to decide about a distribution. In our study, the children behaved notably different when choosing a procedure compared to when directly choosing a distribution. Even three-year-olds are able to share the chances of getting a big resource by using a fair procedure. In fact one five-year-old girl tried to use a procedure to solve her inner conflict in the distribution choice task. She debated the options and then wanted to use a counting rhyme. When the experimenter asked her to just pick one option she took the unfair one. Especially in the older age group the majority of the children chose the fair option either only in the procedure choice task or in both tasks. But just a few children chose the fair option solely in the distribution choice task while being selfish in the procedure choice task. This could imply that it is easier for children to be fair when a decision-making procedure is intermitted and allows them to stand back from their urging desire to gain as much as possible.

Another aspect we manipulated was whether the other receivers were present or not while the child was making a decision. The literature suggests that the presence of affected play partners can influence fairness-related behavior in two directions: It has been shown in several studies that a present and passive receiver promotes anti-social tendencies like Schadenfreude or envy [35], spite [24, 36], and competition [11], which would lead to less sharing when a decision is made in public. However, an empathy-based and also a strategic approach would predict a different behavior: With regard to empathy, a present receiver might be more likely to be considered when a child decides whether to share or not. In the later preschool years, children’s empathy predicts their willingness to share [37] and it is conceivable that it is easier to empathize with someone in face-to-face interaction. Moreover, it can also be beneficial to follow the rather strategic motivation to establish a reputation of being a fair cooperator by sharing when being observed by others [22, 23]–especially when the observer is a potential reciprocator [38]. However, it is important to note that sharing in these studies was not costly and children were explicitly told that the observer would later decide whether she wants to share another (more valuable) resource in return. In another study without these features (i.e. no costly sharing, no direct or indirect reciprocity) by Buhrmester, Goldfarb [39] conducted with five-, nine- and thirteen-year-olds, even the youngest participants shared more resources in a public than in a private situation. In our task, however, we observed neither a decrease nor an increase in sharing, possibly because the children did not really care about the presence of the other children. Another explanation would be that some kids were affected in one direction (being nicer) and others in the other (being less nice) and they evened out in the results. A within-subjects design in which the very same children are tested in private and in a public condition could answer the question why we did not find a difference between these two conditions in our study. Another possible reason might be that the participant possibly expected the other group members to find out later about the number of resources she won (hence the private condition did not really ‘feel’ private). But the mere outcome would not reveal the specific procedure choices which led to it, since it is also possible to just be lucky and win the big sticker box with a fair procedure. Shaw, Montinari [40] showed that children are able to use a fair decision-making procedure (i.e. acting as if they would flip a coin to make a fair decision) as a cover for cheating (just assigning the better price to themselves or flipping the coin until the desired outcome came up). The children in our study could have done the same in the private condition and make unfair decisions knowing that the other (absent) participants would not find out. This would have led to higher rates of unfair decisions in the private condition compared to the public condition. However, the children we tested did not do so in our task.

Our study is the first one investigating both procedural and distributive justice concerns in preschoolers in a group context. It is also the first study to directly manipulate the two important social features of ‘affectedness’ and ‘presence of play partners’ in both situations, which is important for interpreting children’s fairness-related behavior. The results show that the preschoolers in our study were not willing to directly sacrifice a certain resource for the sake of the group, which indicates selfish tendencies typical of that age. However, when using a procedure to distribute the resources even the younger children were sensitive to the affectedness of others by their decision and chose the selfish option less often than when they were playing alone, regardless of whether the others were present or not. Decision-making procedures can help to avoid or solve conflicts of interest within a group. Children are able to recognize and appreciate that even before they develop a clear preference for distributive justice when it is costly to them. This is in line with the more recent literature (e.g. [41]) suggesting that the human concern for fairness needs to be defined in a broader way than just as a preference for equal outcomes.

## Supporting information

S1 Table Raw data for main analysis including children’s choices in the two tasks. (XLSX)
